# Finger Joints Reconstructive Coverage with Cross-Arm (Colson) Flaps After Burn Injury: A Literature Review and Our Experience

**DOI:** 10.3390/healthcare13233114

**Published:** 2025-12-01

**Authors:** Ziyad Alharbi, Maysaa Alghamdi, Johannes Hertelendy, Khalid Khatib, Norbert Pallua

**Affiliations:** 1 Plastic Surgery and Burn Unit, Dr. Soliman Fakeeh Hospital, Jeddah 21461, Saudi Arabia; 2 Clinical Sciences Department, Fakeeh College for Medical Sciences, Jeddah 21461, Saudi Arabia; 3 College of Medicine, University of Jeddah, Jeddah 23218, Saudi Arabia; 4 Department of Plastic and Reconstructive Surgery, Florence-Nightingale Hospital, 40489 Düsseldorf, Germany; 5 Department of Plastic Surgery, RWTH Aachen University Hospital, 52074 Aachen, Germany; 6 Plastic Surgery Elite, Pallua Clinic, 40212 Düsseldorf, Germany

**Keywords:** pedicled flap, upper arm, burn injury, skin defect, hand injuries, finger injuries, random pattern flap, upper arm flap, groin flap

## Abstract

**Background:** Random pattern flaps are widely used in reconstructive surgery when inadequate vascularity precludes skin graft survival or when regional pedicled flaps are unavailable due to local burn injury. Here, thin tissue from the upper arm was utilized to cover exposed cartilage over the proximal interphalangeal (PIP) joints of the contralateral hand. **Methods/Technical Note:** We report the uncommon application of multiple cross-arm (Colson) flaps to reconstruct dorsal skin defects over the PIP joints of the index, middle, and ring fingers following a high-voltage burn injury, in conjunction with a comprehensive literature review. **Results:** Three separate random-pattern flaps were harvested from the upper arm and transferred to the contralateral hand. All flaps demonstrated good perfusion, durable coverage, and a clean wound bed postoperatively, with preservation of joint mobility. **Conclusions:** To our knowledge, this represents one of the first reported reconstructions of multiple adjacent PIP joints using individual cross-arm flaps. This technique remains a dependable salvage option that should be considered in complex reconstructive scenarios when local or microsurgical options are not feasible.

## 1. Introduction

Injuries of the hand can be broadly classified into acute traumatic injuries—such as burns, lacerations, and crush injuries—and chronic or sequelae-related conditions, including contractures and post-burn deformities. Burn injuries themselves vary widely in mechanism, depth, and severity, and can be classified both by etiology (thermal, electrical, chemical, radiation) and by depth of tissue injury. Superficial burns involve only the epidermis and generally heal without surgery, whereas partial- and full-thickness burns extend deeper into the dermis, often requiring surgical intervention and leaving permanent scarring; fourth-degree burns reach underlying muscle or bone, leading to necrosis and loss of function. Importantly, while thermal burns are usually more apparent and superficial, electrical burns may cause disproportionately deep destruction with minimal external signs, as current travels through high-resistance tissues such as muscle, tendon, and bone. This concealed but severe injury is particularly critical in functionally complex regions like the hand, where early recognition and a specialized reconstructive strategy are essential to restore function and minimize long-term disability [1].

To understand the unique clinical behavior of electrical injuries, it is essential to consider their underlying pathophysiologic mechanisms. These injuries involve a combination of thermal and non-thermal damage. Brief exposure may lead to electroporation-induced membrane disruption, while prolonged contact results in Joule heating and widespread cellular necrosis. Despite potentially normal skin appearance, deeper tissues—including tendons, cartilage, and neurovascular structures—may be extensively damaged. In the hand, where soft tissue is limited, such deep injuries present a major reconstructive challenge. When skin grafts fail due to avascular wound beds, vascularized flap coverage becomes essential to restore function and prevent infection [2].

A comprehensive review analyzing 5485 adult electrical injuries worldwide (2017) highlights critical differences between low-voltage (<1000 V) and high-voltage (>1000 V) burns. High-voltage injuries are associated with significantly higher rates of morbidity and mortality: surgical intervention was required in nearly 80% of cases, compared to just over 54% for low-voltage injuries. Amputations occurred in approximately 30% of high-voltage cases versus only 7% in low-voltage situations. Additionally, high-voltage burns were linked to increased compartment syndromes, fasciotomies (27% vs. ~5%), and longer hospital stays—an average of 31 days compared to 11 days for low voltage. Mortality was also doubled (5.2% vs. 2.6%) in high-voltage injuries. These findings demonstrate that high-voltage electrical burns cause far more extensive deep tissue damage and functional compromise than low-voltage injuries, underscoring the necessity for aggressive surgical debridement and early reconstructive interventions [3].

Contemporary burn management emphasizes a multidisciplinary and individualized approach, determined by burn depth, total body surface area (TBSA), and underlying tissue involvement. While superficial burns may be treated conservatively with topical therapies and skin grafting, deeper injuries—particularly high-voltage electrical burns involving muscles, tendons, or cartilage—often necessitate early surgical intervention. A large-scale study analyzing 595 electrical burn cases reinforced this need, highlighting the importance of early fluid resuscitation, fasciotomy, and staged debridement within the first 72 h. Notably, approximately 64% of patients required surgical management, including grafting, flap coverage, or even amputation. These findings reflect the limitations of grafting alone in deep burns and support the shift toward vascularized flap reconstruction when structural tissues are exposed [4,5].

Following wound bed preparation, reconstruction of burn-related defects may be achieved using local or regional flaps, with the choice determined by patient stability, defect complexity, and donor site availability. A flap is defined as the transfer of tissue with its intrinsic blood supply, classified by vascular pattern, proximity to the defect, tissue composition, and method of transfer. Among these, random skin flaps are composed of skin and/or subcutaneous tissue, with vascularity derived from the subdermal plexus rather than a named axial vessel. As local flaps designed adjacent to the defect, their survival depends on maintaining an adequate length-to-width ratio. Random flaps are particularly suited for small- to medium-sized defects where local tissue is available, and they remain widely favored for their simplicity, reliability, and ease of execution in reconstructive surgery. In functionally critical regions such as the hand, optimal outcomes depend on timely, multidisciplinary care, making flap-based reconstruction a cornerstone of management in complex burn cases [5,6]. (see Table 1).

The cross-arm (Colson) flap, first described in the 1970s, provided a reliable method for transferring vascularized tissue to the injured hand [7]. However, its use has declined in the modern reconstructive era due to the availability of microsurgical free flaps and perforator-based options, which allow thinner coverage and earlier mobilization [8]. The cross-arm flap is further limited by the need for staged division, temporary bilateral immobilization, and its unsuitability in bilateral injuries, making it less favored in current practice [9]. Nevertheless, it remains a pragmatic salvage option when microsurgical expertise is unavailable or contraindicated.

This review and technical note highlight the effective use of multiple cross-arm random pattern flaps in reconstructing dorsal finger defects following high-voltage electrical burn injury—a technique that remains underutilized in modern reconstructive practice. The review aims to contribute to the limited literature supporting this approach in cases where conventional methods fail due to extensive tissue damage.

## 2. Method and Technical Note

**Figure 1:**
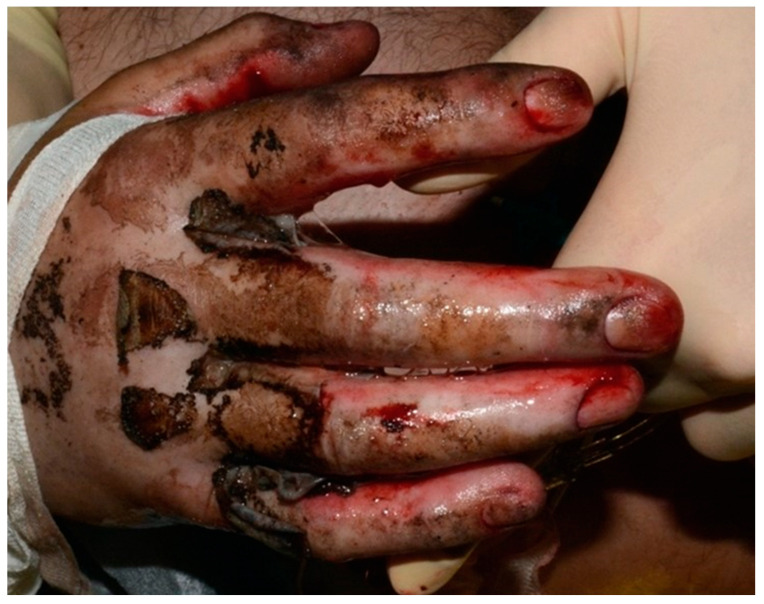
This picture shows the initial presentation of the third-degree burns over the joint areas.

**Figure 2:**
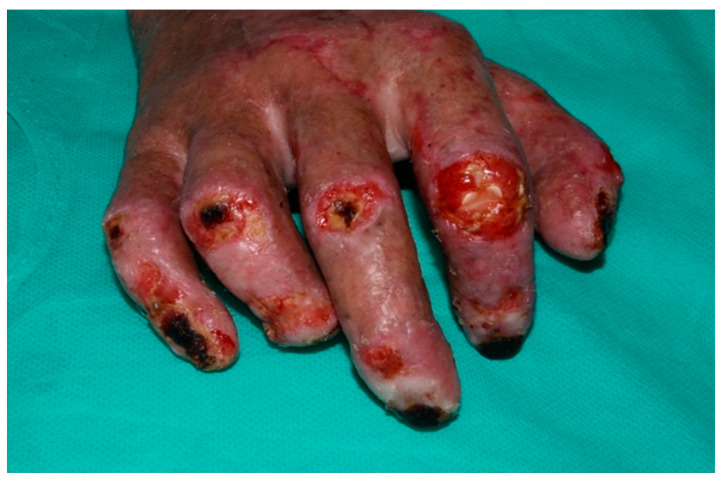
This picture shows the first postoperative situation where PIP joints were exposed.

Following stabilization, a patient with a third-degree high-voltage burn injury involving more than 50% of the total body surface area (TBSA) and complicated by joint exposure was admitted directly to the intensive care burn unit (ICU), where an aggressive multidisciplinary management plan was promptly initiated (Figure 1). Initial treatment included thermal cleansing and staged surgical intervention beginning with debridement of necrotic tissue and urgent escharotomy to relieve pressure and prevent compartment syndrome in the affected limbs. In the subsequent days, two additional debridement procedures were required to achieve adequate clearance of devitalized tissue. To optimize wound healing and minimize the risk of infection, xenografts (MatriDerm^®^) were applied to the wound bed between procedures, serving as temporary biological coverings and aiding in preparation for definitive reconstruction. After the second debridement, split-thickness skin grafting (STSG) was performed to cover burn wounds on the upper limbs. The grafts successfully integrated in most areas. However, on the dorsum of the right hand—specifically over the second, third, and fourth proximal interphalangeal (PIP) joints—graft failure occurred (Figure 2). These areas exhibited full-thickness tissue loss with exposed tendons and cartilage, conditions known to impede graft take due to poor vascularization.

Given the inability of traditional STSGs to adhere in these regions of exposed cartilage and tendon, an alternative reconstructive strategy was deemed necessary. A secondary attempt at coverage using dermal skin substitutes (MatriDerm^®^) also failed, further confirming the unsuitability of grafting techniques in this particular context. The decision to proceed with vascularized flap coverage was based on several considerations: the critical functional role of the PIP joints, the deep nature of the injury, and the risk of infection or joint contracture if left uncovered. Local or regional flap options were contraindicated due to the extent of ipsilateral upper extremity burns, while free flap reconstruction was considered less feasible given the patient’s systemic condition and extensive TBSA involvement. Ultimately, the surgical team elected to harvest three thin random-pattern flaps from the contralateral upper arm to cover each of the three dorsal PIP joint defects individually. This approach aimed to provide well-vascularized tissue from an uninjured donor site while preserving joint mobility and minimizing the risk of infection.

**Figure 3:**
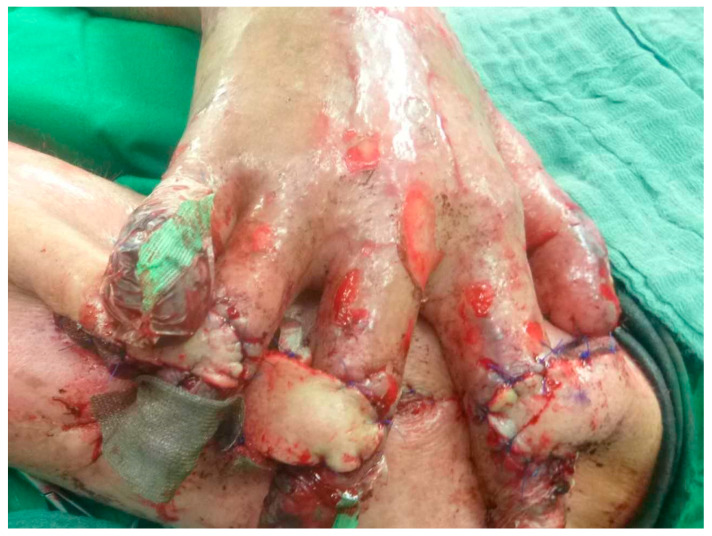
This picture shows the inset of the Colson Cross-Arm Flap (contra lateral side) over the exposed PIP joints.

**Figure 4:**
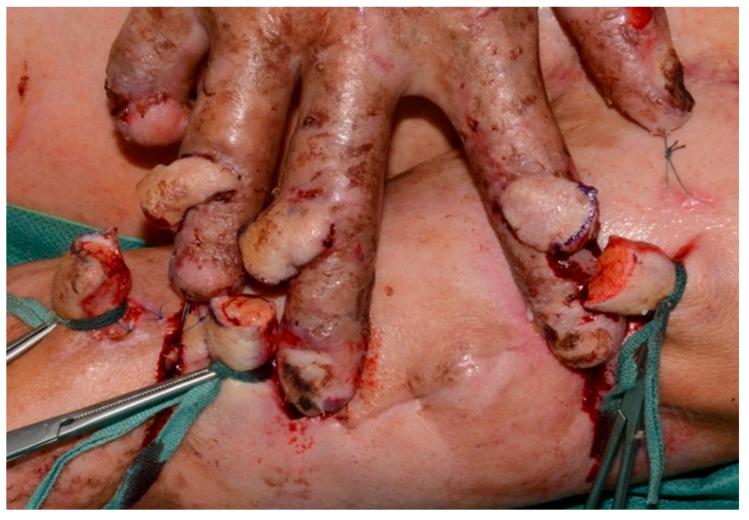
This picture shows the separation of pedicles of the Colson Cross-Arm Flap over the exposed PIP joints with good vascular supply from the wound grounds.

**Figure 5:**
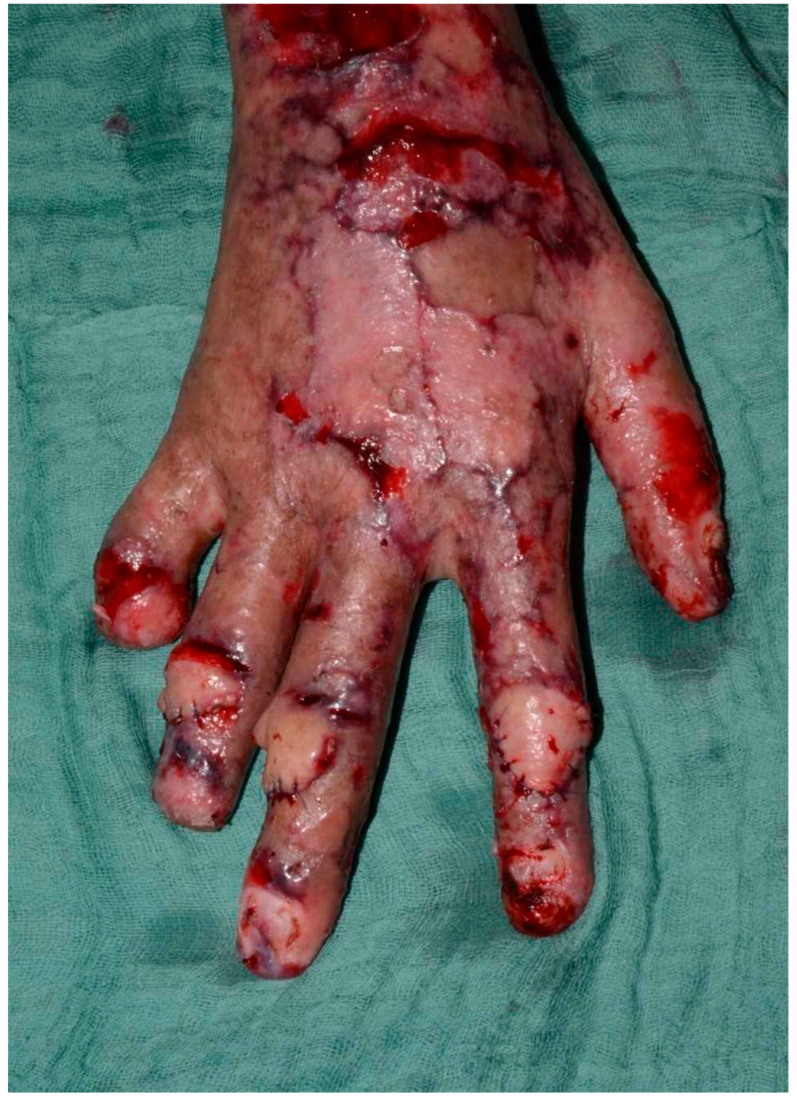
This picture shows the complete healing of the Colson Cross-Arm Flap over the exposed PIP joints during the care phase.

During surgery, the three random-pattern flaps were elevated from the medial aspect of the left upper arm and transposed across to the right hand in a cross-arm configuration (Figure 3 and Figure 4). The design permitted tension-free inset of each flap over the respective PIP joint defect by tailoring the flap size appropriately, thereby ensuring coverage of exposed cartilage with pliable, well-vascularized tissue. Postoperative care involved specialized pressure-relieving dressings, pain management with analgesia and sedation, immobilization inside the hospital and initiation of flap training on postoperative day ten. This process involved gradual occlusion of the pedicle for increasing time intervals, starting from 5 min and extending to 45 min daily, to promote neovascularization from the recipient bed. The flaps remained well perfused throughout the 14-day postoperative period, with no evidence of infection or ischemic compromise (Figure 5). On day 14, the patient was taken back to the operating theater for pedicle separation. We followed the standard timeframe of 14 to 21 days to ensure that the flaps were well perfused prior to division. After confirming adequate perfusion, all pedicles were separated, and the edges were sutured with Prolene sutures, which was removed 14 days later. The donor site was also repaired using the remaining skin and closed accordingly. The patient recovered without complications, was transferred to the general ward in stable condition, and was discharged to a rehabilitation center following flap separation and complete wound healing. Importantly, the patient expressed significant satisfaction with both the esthetic outcome and restored hand function. He noted initial concerns about the immobilization period, but later described the surgical outcome as transformative in terms of independence, daily task performance, and overall quality of life.

## 3. Discussion

Electrical burn injuries involving the hand pose a significant reconstructive challenge, particularly when deep structures such as tendons, cartilage, and joints are exposed. Cross-arm flaps have been reported in limited series, including the use of eight fasciocutaneous and forearm pedicled flaps in three patients with reliable coverage and satisfactory function [10]. Similarly, older reports have described cross-arm dermis flaps for distal dorsal finger defects with encouraging outcomes [11]. However, their application to multiple adjacent finger joints has rarely been documented. To our knowledge, reconstruction of three dorsal PIP joints using separate random-pattern cross-arm flaps from the contralateral limb has not been previously reported. Our case therefore highlights an uncommon but practical adaptation of an established technique, providing thin vascularized coverage from an uninjured donor site and preserving joint mobility in the setting of extensive tissue loss and limited local options.

Our case parallels the experience described by Shah et al., who in 2019 managed a 43-year-old female patient with bilateral high-voltage electrical burns to the hands using bilateral cross-arm pedicled flaps [9]. In that case, both thumbs were successfully resurfaced with forearm-based random pattern flaps, resulting in excellent perfusion, early mobilization, and complete return to pre-injury activities—including high-demand functions such as rock climbing. Their report highlights the feasibility and functional benefit of using distant pedicled flaps in complex hand injuries [9]. Similarly, our case demonstrates that applying three separate contralateral upper arm flaps can offer a reliable, anatomically appropriate, and function-preserving solution in patients with extensive dorsal finger defects and limited donor site availability.

While numerous reconstructive options exist for digital soft tissue defects, patients with extensive burns and high TBSA involvement often have limited choices. Local and regional pedicled flaps are generally contraindicated when the ipsilateral extremity is affected by burns. Free flaps, though viable, are resource-intensive, require microsurgical expertise, and may not be feasible in critically ill patients [12]. A study by Goertz et al. on pedicled groin flaps demonstrated good results in hand defect coverage, but the bulkiness and discomfort associated with groin tissue, along with restrictions on patient mobility, remain concerns [13]. Wu et al. described successful use of abdominal flaps in reconstructing deep digital burns; however, the technique demands prolonged immobilization and patient cooperation, which can be difficult in multi-site injuries [14].

The medial upper arm fasciocutaneous flap, first introduced by Bhattacharya et al., offers a thinner and more pliable option for covering hand and distal forearm defects, with reduced donor site morbidity and enhanced postoperative comfort compared to bulkier alternatives [15]. This technique provides reliable vascularity and is particularly advantageous in patients where donor site flexibility and minimal immobilization are crucial. Building upon earlier foundational work by Dolich et al. and Dickinson et al., who pioneered and refined cross-arm flaps in complex hand reconstruction, our case applies this concept in a novel context—reconstructing exposed cartilage over the dorsal PIP joints following high-voltage electrical burns [7,16]. By using three contralateral upper arm random-pattern flaps, we achieved stable, tension-free coverage while preserving function. Despite the challenge of temporary bilateral upper limb immobilization, this approach proved effective in addressing extensive tissue loss when conventional methods were unsuitable.

Management of high-voltage electrical burn injuries to the hand begins with early and repeated debridement to remove devitalized tissue and prevent progressive necrosis [4]. Once a viable bed is established, reconstructive options are selected according to the depth and extent of injury. Adjunctive measures such as negative-pressure wound therapy can optimize wound beds and temporize coverage, although they are insufficient as a definitive reconstructive solution [4,5]. Conventional skin grafting, while simple and widely available, is particularly limited in deep burns: it provides only superficial coverage, lacks vascularity, contracts significantly over time, and fails to protect underlying structures such as exposed bone, tendon, or cartilage, making it unsuitable for long-term functional preservation [17]. Dermal substitutes such as Integra, when staged with full-thickness skin grafting, provide stable coverage and help mitigate re-contracture, though their cost, availability, and poor durability over exposed cartilage limit broader use [18]. Local and regional flaps, including cross-finger, groin, or perforator-based propeller flaps, yield thin, reliable coverage with preservation of axial vessels, but their success requires intact perforators and microsurgical expertise, rendering them unsuitable in heavily scarred or electrically injured fields [19]. Local double-opposing rearrangements like the five-flap Z-plasty are effective for superficial, linear PIP contractures but do not address deeper tissue loss [20]. Free-tissue transfer remains the gold standard for extensive or complex injuries, offering robust vascularized coverage, yet it demands microsurgical expertise, prolonged operative time, and a stable systemic condition—factors often compromised in high-voltage electrical injuries [8]. Adjunctive modalities such as autologous fat grafting or cell-assisted lipotransfer may improve scar pliability and pain, but they are insufficient for primary joint or cartilage coverage [21]. Cellular and engineered matrices have demonstrated encouraging results across wound types with low infection rates and improved pliability, yet they cannot replace vascularized flap coverage in defects with exposed bone or cartilage [22].

In contrast, the cross-arm flap provides thin, vascularized coverage through a technically straightforward, reliable approach that does not require microsurgery. Its drawbacks—most notably temporary bilateral immobilization, staged division, and unsuitability in bilateral injuries—translate into a high patient burden, as weeks of immobilization can compromise self-care, mobility, and overall quality of life. Mitigation strategies include careful preoperative counseling, provision of nursing and family support during immobilization, and functional splinting to minimize discomfort; importantly, early physiotherapy after flap division is essential to restore mobility and reduce stiffness. Standard rehabilitation protocols emphasize supervised mobilization soon after flap separation and wound healing (21 days), progressive strengthening, and joint-specific exercises to maintain tendon gliding and prevent contracture [23]. Long-term functional outcomes are best assessed through range-of-motion and grip strength testing, together with patient-reported scores such as the Disabilities of the Arm, Shoulder and Hand (DASH) or Michigan Hand Questionnaire (MHQ) [24,25]. In our patient, extensive ipsilateral scarring and exposed dorsal PIP cartilage made local and microsurgical options unfeasible, supporting the cross-arm flap as the most pragmatic and dependable solution in this unique context.

Despite these strengths, it is important to acknowledge the broader limitations of this work. Scientifically, the cross-arm flap is not novel, having been established in the 1970s; performing three simultaneous flaps does not itself constitute a technical innovation. The contribution of this case lies instead in illustrating that, even in the modern reconstructive era, this older technique remains clinically relevant when newer options are unavailable or unsuitable. Furthermore, cross-arm flaps inherently require careful postoperative monitoring to safeguard vascular integrity and functional recovery, and long-term follow-up is needed to assess joint mobility and scar quality. Importantly, this technique is not universally applicable, and careful patient selection is mandatory to ensure feasibility and safety. Future studies and larger case series may help refine patient selection and optimize rehabilitation protocols to maximize functional outcomes.

## 4. Conclusions

This literature review and technical note demonstrate that cross-arm (Colson) flaps, though less frequently used in contemporary reconstructive practice, remain a vital salvage option for complex high-voltage electrical burn injuries when modern alternatives are not feasible. In our patient, three simultaneous random-pattern flaps provided durable wound healing, preserved dorsal PIP joint mobility, and maintained satisfactory hand function at follow-up. The central take-home message is that traditional pedicled flaps continue to offer reliable, practical solutions in resource-limited or clinically challenging scenarios. Looking ahead, prospective case series and multi-center collaborations are warranted to systematically evaluate long-term functional outcomes, complication profiles, and patient-reported satisfaction, thereby better defining the role of cross-arm flaps in modern reconstructive surgery.

## Figures and Tables

**Table 1:** Comparison of reconstructive coverage options for burn injuries.

Coverage Options	Skin Layers Included	Indications	Advantages	Limitations	Wound Healing Outcomes	Complications
Skin grafts (split- or full-thickness)	STSG = epidermis + part of dermis FTSG = epidermis + entire dermis	Superficial or intermediate burns, vascularized wound beds	Simple, rapid coverage, widely available, minimal donor morbidity	Fail on exposed tendon, bone, or cartilage; secondary contracture; no intrinsic vascularity	Bad	Graft loss, infection, hypertrophic scarring, contracture
Dermal scaffolds (e.g., Integra, collagen–elastin matrix)	Acellular dermal matrix substitute requires epidermal coverage	Deep burns with partial exposure; staged use before grafting	Improves pliability, reduces contracture, provides neodermis	Expensive, limited availability, poor durability over bare bone or cartilage, infection risk	Good	Infection, delayed vascularization, scaffold loss
Skin flaps (local, regional, free, cross-arm)	Epidermis + dermis + subdermal layer (fat)	Deep composite defects with exposed tendon, cartilage, or bone	Vascularized durable coverage; can include multiple tissue types; thin pliable tissue available	Technically more complex; donor-site morbidity; may require microsurgery or immobilization	Great	Partial or total flap loss, venous congestion, donor-site complications, stiffness due to immobilization

## Data Availability

The original contributions presented in this study are included in the article. Further inquiries can be directed to the corresponding author.
